# Expression of concern: An efficient one pot three-component nanocatalyzed synthesis of spiroheterocycles using TiO_2_ nanoparticles as a heterogeneous catalyst

**DOI:** 10.1039/d4ra90021a

**Published:** 2024-03-07

**Authors:** Yogesh Kumar Tailor, Sarita Khandelwal, Yogita Kumari, Kamlendra Awasthi, Mahendra Kumar

**Affiliations:** a Department of Chemistry, University of Rajasthan Jaipur India mahendrakpathak@gmail.com +91 0141 2702720; b Soft Materials Lab, Department of Physics, Malaviya National Institute of Technology Jaipur India

## Abstract

Expression of concern for ‘An efficient one pot three-component nanocatalyzed synthesis of spiroheterocycles using TiO_2_ nanoparticles as a heterogeneous catalyst’ by Yogesh Kumar Tailor *et al.*, *RSC Adv.*, 2015, **5**, 46415–46422, https://doi.org/10.1039/C5RA04863J.

In the original article, the authors recognise concerns with the integration values in the ^1^H NMR spectra for compounds **4e**, **4j**, **4l**, **4n**, **5e**, **5j**, **5l**, **5m** and **5n**. The integration values for these compounds do not match the reported structures. This is currently under consideration, but in its current form, the ^1^H NMR data for these compounds should not be considered to be correct.

An update will be provided as soon as possible.

Dr Laura Fisher

1st March 2024

Executive Editor, *RSC Advances*

## Supplementary Material

